# Supplementary material for: Adherence to the Japanese Food Guide: The Association between Three Scoring Systems and Cardiometabolic Risks in Japanese Adolescents
Source: Nutrients. 2021 Dec 23;14(1):43. doi: 10.3390/nu14010043 (PMC8746488; doi:10.3390/nu14010043)
Supplement: Supplementary file 1 [file nutrients-14-00043-s001.zip › nutrients-1514652-supplementary.pdf]

Table S1. Food groups in the Japanese Food Guide Spinning Top and their optimal ranges.

Food groups	Food items of the BDHQ	ORG score		MOD1 score		MOD2 score	
		Female	Male	Female	Male	Female	Male
Grain dishes	Rice, bread, buckwheat noodle, Japanese wheat noodle, Chinese wheat noodle, and pasta and macaroni (6 items).	5–7 SV	6–8 SV	5–7 SV	6–8 SV	5– SV	6– SV
Vegetable dishes	Salted green and yellow pickled vegetable; other salted vegetables; raw vegetables used in salad; green leafy vegetables including broccoli; cabbage and Chinese cabbage; carrots and pumpkins; radishes and turnips; other root vegetables; tomatoes; mushrooms; seaweeds; French fries; sweat, yams and taro potatoes; and vegetable juice (14 items).	5–6 SV	6–7 SV	5– SV	6– SV	5– SV	6– SV
Fish and meat dishes	Chicken; pork and beef; ham, sausages, and bacon; liver; squid, octopus, shrimp, and clam; small fish with bones; canned tuna; dried fish and salted fish; oily fish; non-oily fish; fish paste; eggs; tofu and tofu products; and natto (14 items).	3–5 SV	4–6 SV	3–5 SV	4–6 SV	3– SV	4– SV
Milk	Low-fat milk, full-fat milk, yogurt, and cheese (4 items).	2 SV	2–3 SV	2 SV	2–3 SV	2- SV	2– SV
Fruits	Citrus fruit; strawberries, persimmons, and kiwi fruits; and other fruits (3 items).	2 SV	2–3 SV	2- SV	2– SV	2- SV	2– SV
Sugar and confectionaries	Cakes, cookies, and biscuits; Japanese sweets; rice crackers; ice cream; cola and sugar-sweetened soft drinks; snack foods; chocolate; jam and marmalade; Lactobacillus beverages; sugar for coffee and black teas; nutritional supplement; nutritional supplement drinks (12 items).	< 200 kcal	< 200 kcal	< 200 kcal	< 200 kcal	< 200 kcal	< 200 kcal

ORG score, original score. MOD1 (first modified) score was calculated as scores with no upper limits for vegetable dishes and fruits. MOD2 (second modified) score was calculated as scores with no upper limits for all dishes except for sugar and confectionaries. Energy requirements for adolescents are 2000–2400 kcal for females, and 2400–3000 kcal for males. One serving (SV) corresponds to 40 g of carbohydrate for grain dishes, 70 g of vegetables for vegetable dishes, 6 g of protein for fish and meat dishes, 100 mg of calcium for milk, and 100 g of fruits for fruits. The number of servings was rounded to whole numbers as follows: if the value obtained is between 0.67 and < 1.5, it was counted as one serving; values between 1.5 and < 2.5 times was rounded off to two servings, values between 2.5 and < 3.5 times was rounded off to three servings. The rounding-off manner applies to the succeeding values (i.e., 3.5 to <4.5 and so on). For each dish, the maximum point is 10. When the intake for former five food groups is lower or higher than the optimum, a point is calculated as SV/lower limit of the optimum × 10 or 10 − (SV − the upper limit)/ upper limit × 10, respectively. When the calculated point is less than zero, no point is given. Optimum intake of sugar and confectionaries is < 200 kcal giving 10 points, and points for ≥ 200 kcal is calculated as (corresponding energy − 200)/ 200 × 10.

1. Yoshiike N,; Hayashi F,; Takemi Y, ; Mizoguchi K,; Seino F. A new food guide in Japan: the Japanese food guide Spinning Top. *Nutr Rev* **2007**, 65(4):149-54. doi: 10.1111/j.1753-4887.2007.tb00294.x.

2. Ministry of Health Laboror and Wealfare. Report on Utilization of Dietary Reference Intakes for Japanese Tokyo: Ministry of Health Labour and Welfare, **2010**. Available online: https://www.mhlw.go.jp/shingi/2010/03/s0331-9.html (accessed on Oct. 25, 2021).

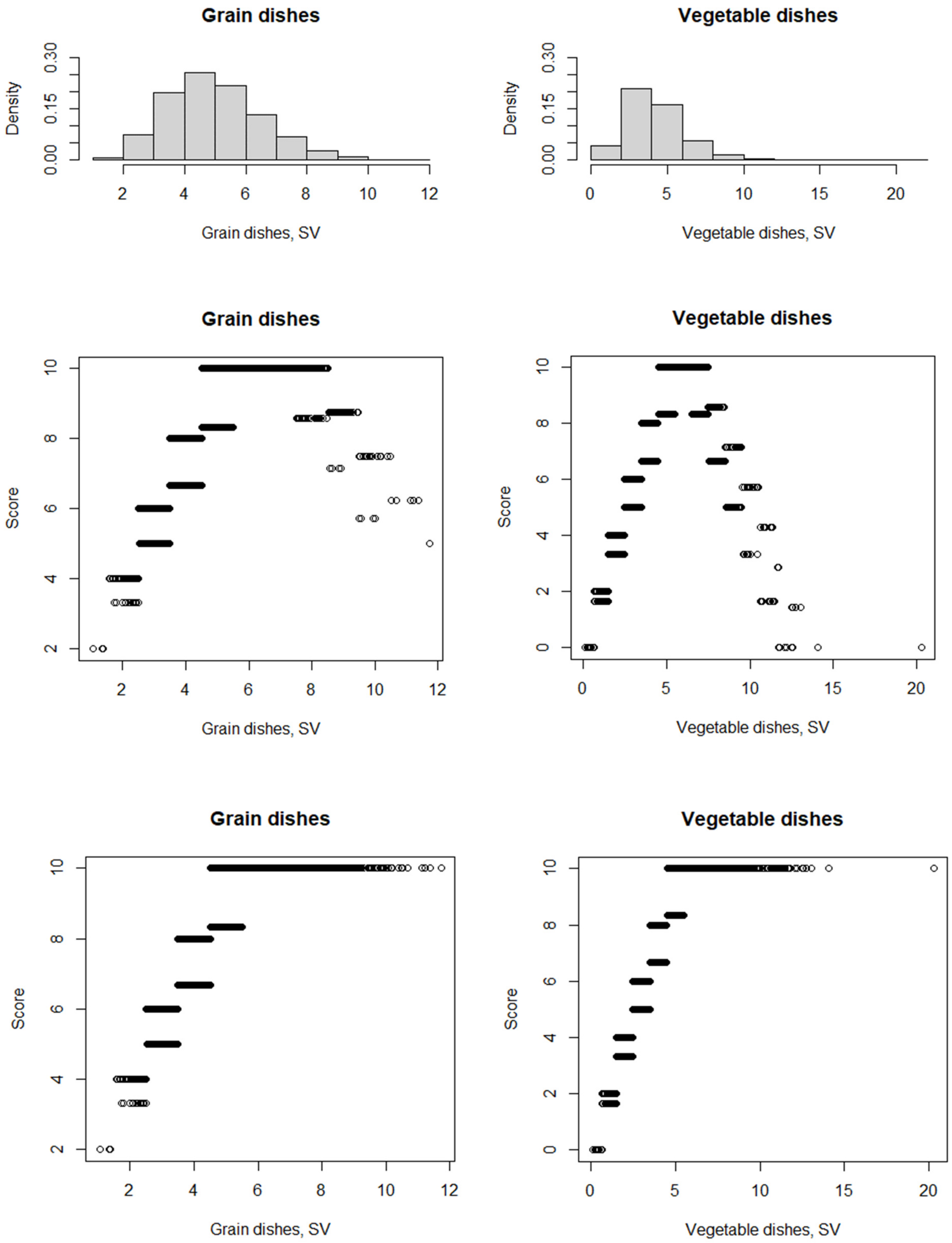

Figure S1. Histogram and scores for grain and vegetable dishes.

Upper panels, density histogram of servings (SV); middle panels, scores with lower and upper limits; lower panels, scores with lower limits, and without upper limits.

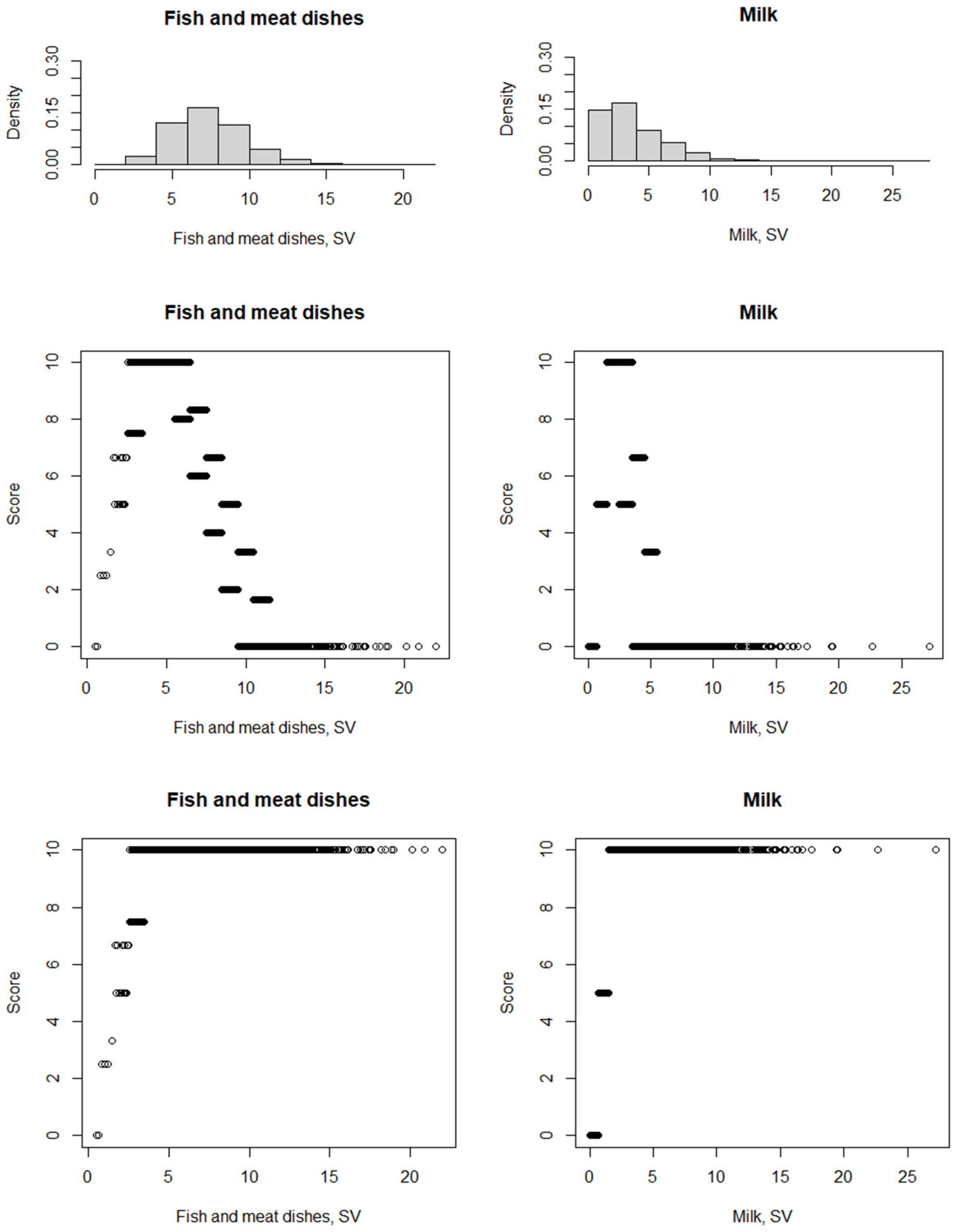

Figure S2. Histogram and scores for fish and meat dishes and milk.

Upper panels, density histogram of servings (SV); middle panels, scores with lower and upper limits; lower panels, scores with lower limits, and without upper limits.

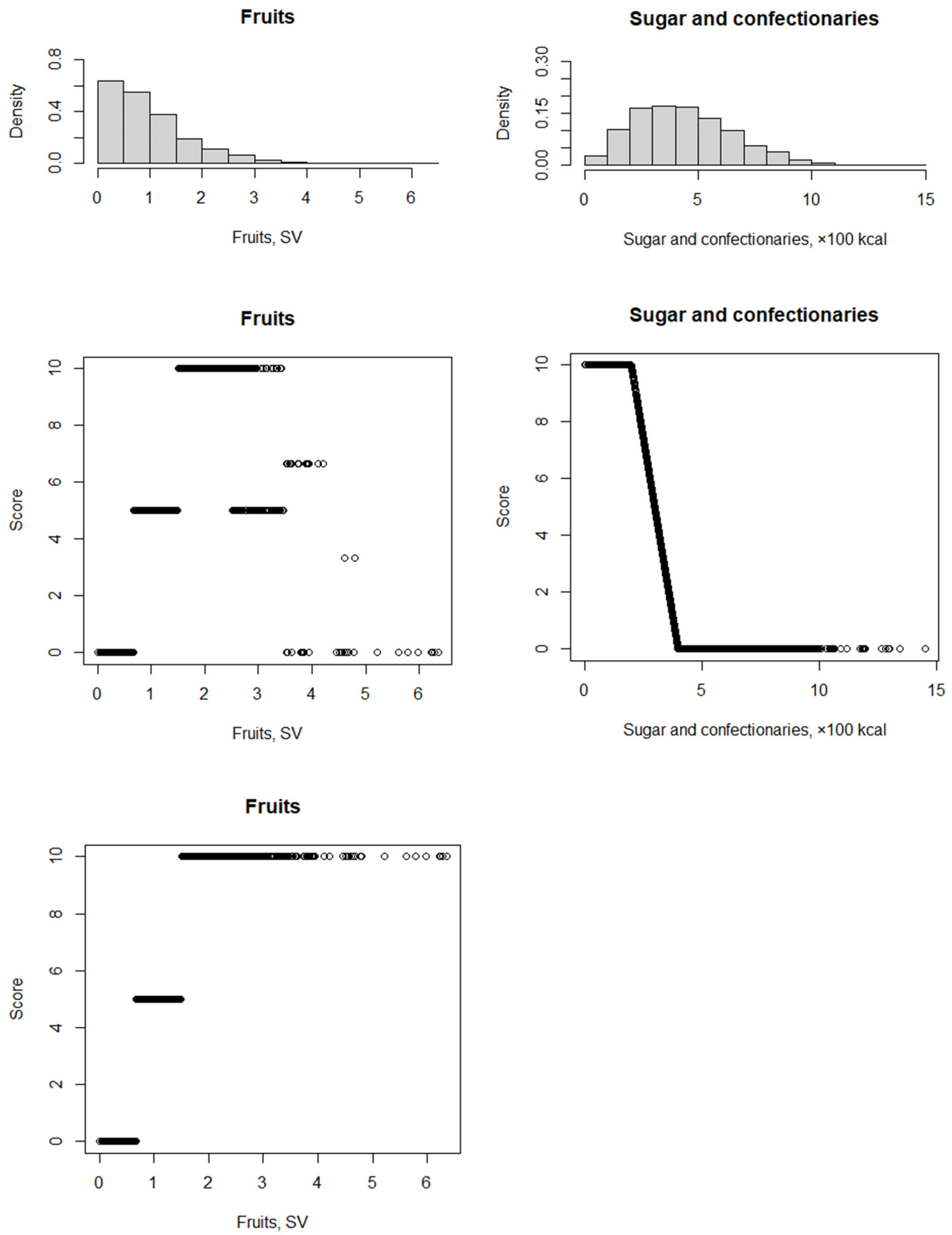

Figure S3. Histogram and scores for fruits and sugar and confectionaries.

Upper panels, density histogram of servings (SV); middle panels, scores with lower and upper limits for fruits, and scores with upper limits for sugar and confectionaries; lower panels, scores with lower limits, and without upper limits.
